# Alpha-tryptase gene variation is associated with levels of circulating IgE and lung function in asthma

**DOI:** 10.1111/cea.12259

**Published:** 2013-05-24

**Authors:** A M Abdelmotelb, M J Rose-Zerilli, S J Barton, S T Holgate, A F Walls, J W Holloway

**Affiliations:** 1 Clinical and Experimental Sciences Unit, Faculty of Medicine, University of SouthamptonSouthampton, UK; 2 Faculty of Medicine, Tanta UniversityTanta, Egypt; 3 Human Development and Health Unit, Faculty of Medicine, University of SouthamptonSouthampton, UK; 4 MRC Life Course Epidemiology Unit, Faculty of Medicine, University of SouthamptonSouthampton, UK

**Keywords:** asthma, gene copy number variation, IgE, mast cell, tryptase

## Abstract

**Background:**

Tryptase, a major secretory product of human mast cells has been implicated as a key mediator of allergic inflammation. Genetic variation in the tryptases is extensive, and α-tryptase, an allelic variant of the more extensively studied β-tryptase, is absent in substantial numbers of the general population. The degree to which α-tryptase expression may be associated with asthma has not been studied. We have investigated the α-tryptase gene copy number variation and its potential associations with phenotypes of asthma.

**Objectives:**

Caucasian families (*n* = 341) with at least two asthmatic siblings (*n* = 1350) were genotyped for the α-tryptase alleles, using high-resolution melting assays. Standards for the possible α-/β-tryptase ratios were constructed by cloning α-and β-tryptase PCR products to generate artificial templates. Association analysis of asthma affection status and related phenotypes [total and allergen-specific serum IgE, bronchial hyperresponsiveness to methacholine, forced expiratory volume in 1s (FEV_1_) and atopy and asthma severity scores] was undertaken using family-based association tests (FBAT).

**Results:**

Four consistent melting patterns for the α-tryptase genotype were identified with alleles carrying null, one or two copies of the α-tryptase allele. Possessing one copy of α-tryptase was significantly associated with lower serum levels of total and dust mite-specific IgE levels and higher FEV_1_ measurements, while two copies were related to higher serum concentrations of total and dust mite-specific IgE and greater atopy severity scores.

**Conclusions and Clinical Relevance:**

Associations of α-tryptase copy number with serum IgE levels, atopy scores and bronchial function may reflect roles for tryptases in regulating IgE production and other processes in asthma.

## Introduction

The serine protease tryptase has the potential to act as a key mediator in asthma and allergy, and increased levels have been detected in the airways of asthmatics [reviewed in Ref. 1]. A range of functions found for β-tryptase would be consistent with this serine protease contributing to inflammation and tissue remodelling. Thus, it can interact with various cell types, stimulate cytokine release from epithelial 2, endothelial 3,4 and airway smooth muscle cells 5, provoke mast cell degranulation 6,7, induce collagen secretion by fibroblasts 8 and act as a growth factor for fibroblasts 8, airway smooth muscle 5,9 and epithelial cells 2. Injection of human tryptase into animal models can induce microvascular leakage and the accumulation of inflammatory cells 10, while clinical improvement has been reported following administration of inhibitors of tryptase in animal 11–13 and human models of asthma 14. Despite the range of important functions ascribed to tryptase, the architecture of the gene locus remains poorly defined. Little is known of the extent of copy number variation and the potential association with asthma or other conditions.

Although frequently referred to as a single protease, tryptase exists in multiple forms. The best characterised has been termed β-tryptase to distinguish it from the original form to be cloned (subsequently termed α-tryptase) 15. Further sequences derived shortly afterwards 16,17 have been designated β1-, β2- and β3-tryptases. The β-tryptases are considered allelic variants, being 98–99% identical in amino acid sequence. The less closely related α-tryptases are 91% similar at the amino acid level to β-tryptases and have thus been proposed to be a product of a separate gene in the haploid genome 18. The family of tryptases encoded by a region on chromosome 16 comprises also γ, δ and ε forms, but these appear quite distinct in function and genomic location 19. Studies with recombinant preparations of tryptase have indicated that β tryptase is the form released as a major product of mast cell degranulation, whereas α- tryptase is not processed, and as is the case with unprocessed β-tryptase, it is secreted constitutively 20. The observation of cDNA for three variants of β-tryptase in a single donor 17 early raised the prospect of tryptase being encoded by more than one gene. Proposals have included a two-locus model with separate loci for α- and β-tryptases 21, or with α- and β1-tryptases competing allelically at one locus, and β2 and β3 at a the other 19,22. There has been speculation also that there may be a three-locus model with α, β1 and β2 each occupying separate loci 23.

Asthma is a complex multifactorial disease, involving genetic and environmental components to disease expression 24 as well as strong evidence of mast cell and its principal neutral protease, tryptase, being involved in pathogenesis. Recent studies have shown that genetic variation in gene copy number can be associated with disease outcomes through alterations in levels of gene expression 25, although direct associations with asthma have been little studied. Inherited copy number variation has been found to underlie Mendelian diseases in several families 26 and has been suggested to account for some of the missing heritability for common diseases not identified through genome-wide studies of single nucleotide polymorphisms 27. It has recently been reported that copy number variation exists for a number of asthma susceptibility genes, although in most cases they were of low frequency and did not confer a statistical increase in the risk of asthma 28. There is a need to investigate such associations in genes for which copy number variation is more prevalent. The finding that the α-tryptase gene exhibits copy number variation with approximately 29% of Caucasians having no copy of the α-tryptase gene 22 has raised questions as to the potential for α-tryptase expression to be associated with disease.

Given that tryptases may be important inflammatory mediators in asthma, we hypothesized that alteration in α-tryptase copy number would affect susceptibility to allergy and the severity of asthma. To examine this, we have developed a qPCR genotyping assay for the α-tryptase locus. We have investigated variation in copy number in Caucasian families with asthma and investigated associations with asthma and related phenotypes.

## Methods

### Subjects and clinical assessments

Caucasian families (*n* = 341) from the Southampton area were recruited with at least two biological siblings with a current physician diagnosis of asthma and taking medication on a regular basis. Age-adjusted serum total IgE levels and specific IgE levels for grass, house dust mite, cat, dog, *Alternaria* and tree allergens were determined by RAST. Skin prick testing was also completed for the same allergens. Baseline lung function tests (forced expiratory volume in 1s; FEV_1_) were performed. Bronchial hyperresponsiveness (BHR) was measured with the provocation concentration of inhaled methacholine required to reduce FEV_1_ by 20% (PC_20_). An atopy severity score was derived using the first principal component of number and mean weal diameter of positive skin prick responses to allergen and range and level of specific IgE levels. An asthma severity score was derived using the first principal components of BHR, treatment scores based on the British Thoracic Society treatment guidelines and the symptom score derived from a detailed questionnaire of asthma. This population and the generation of the phenotypic scores have been described previously 29–31. Ethical approval was obtained from the Southampton and Southwest Hampshire, and Portsmouth and Southeast Hampshire Joint Ethics Committees.

### PCR genotyping for α-tryptases

Initially, standard PCR was used to generate an assay that could detect the presence or absence of the α-tryptase gene. Given the high homology between all sequences available for both genes, the design of PCR with allele-specific primers was not possible. Therefore, a common amplicon in exon 4 and intron 4 for all α- and β-tryptases sequences deposited in GenBank (AF195508, AF099145, AF099143, AF098328, AF099144, NG_032951 and AF529082) was chosen with specific features for α-tryptase. These were a 10- or 11-bp deletion (compared with β-tryptase) and a single nucleotide difference between α- and β-tryptase sequences that generated a recognition site for *Eco*RV. This results in an amplicon size of 552 bp from β-tryptase and 541–2 bp from α-tryptase and following digestion with *Eco*RV fragments of 552 bp for β-tryptase and 151 and 391 bp for α-tryptase. Primer sequences were TF (5′-GAGTGGGATCCTCCGCTGC-3′) and TR (5′-CGGCACACAGCATGTCGT-3′). Each PCR was carried out in a volume of 10 μL, containing 20 ng DNA, 0.2 mm dNTP, 2 mm MgCl_2_, 0.25 U Taq DNA polymerase (Thermo Scientific, Epsom, UK), primers (0.2 μm of each TF and TR; Eurogentec, Fawley, UK) and 10x standard PCR buffer. The PCR cycling conditions were 2 min at 95°C followed by 35 cycles of 30 s at 95°C, 30 s at 62°C and 37 s at 72°C, and finally, 5 min at 72°C. To check PCR amplification, PCR product was electrophoresed on a 2% agarose gel and visualized with ethidium bromide staining and UV illumination. Following amplification, 20 μL of PCR product was digested in a 10 μL reaction containing 10 U of restriction enzyme *Eco*RV (New England Biolabs) at 37°C for 3 h. Restriction products were electrophoresed in 2% agarose and visualized with ethidium bromide. DNA was extracted from cell lines with known tryptase genotypes for use as controls: HMC-1 (β-tryptase only) and KU812 (α- and β-tryptase) 20,32, and also from LAD2 cells for which the tryptase genotype has not previously been investigated. HMC-1 cells were a kind gift from Dr Joseph H Butterfield (Mayo Clinic, Rochester, MN, USA) and LAD2 cells from Dr Cem Akin (Brigham and Women’s Hospital, Boston MA, USA). KU812 cells were from the European Collection of Animal Cell Cultures (ECACC no 90071804; Salisbury, UK).

### Quantitative copy number assay

To develop an assay more suited to high-throughput genotyping and to give quantitative information on copy number, a high-resolution melt curve assay was established using a pair of primers designed to amplify a common amplicon of 70 bp from both α- and β-tryptase sequences but with six-bp mismatches between them. The final reaction mixture contained 600 nm of each primer [MF (5′-ATCATCGTGCACCCACAGTTCT-3′) and MR (5′-GCTCCTCCAGCTCCAGCAG-3′)], 5 μL of 2X PCR mix (Eurogentec), 1 μL of SYTO09 (Invitrogen, Paisley, UK), 3.8 μL of dH_2_O and 2 μL of template DNA at a concentration of 10 ng/μL. PCR amplification and real-time fluorescent data collection were performed on a LightCycler® 480 equipment (Roche, Hertfordshire, UK); 95°C for 10 min, then 40 cycles of 95°C for 15 s and 60°C for 60 s. Melting profiles were assessed by heating to 95°C for 15 s and then 60°C for 1 s using a temperature transition rate of 4.4°C/s. Control cell line DNA samples were included in each 384-well plate. Derivative melting curves were obtained with LightCycler data analysis software (version 3.5).

### Creation of artificial controls

To validate the copy number variation assay, artificial template controls were generated using the PCR genotyping amplicons. PCR amplification was undertaken as described above but with increased annealing temperature to 65°C and the amplicons visualized on a 3% agarose gel. Appropriately sized products were excised from the gel, purified with a QIAquick Gel Extraction kit (Qiagen, Sussex, UK) and ligated into the pCR®-Blunt vector (Invitrogen). The ligation mixture was used to transform one shot TOP10 *Escherichia coli* cells (Invitrogen). The transformation mixture was plated onto LB/agar plate’s containing kanamycin.

### Screening of plasmid colonies

Plasmids containing the appropriately sized inserts were screened by PCR after RFLP using *Eco*RV to select α-and β-tryptase fragments. Plasmid DNA was then purified using QIAquick miniprep kits (Qiagen), and nucleotide sequencing performed at a core facility (Geneservice, Oxford, UK). DNA copy number was corrected from plasmid to genomic DNA using the equation: number of copies = (amount × 6.022 × 12^23^)/ (length × 1 × 10^9^ × 650) molecules/gram 33. Plasmids containing α- and β-tryptase-specific fragments were mixed in defined proportions to create control templates for different α-tryptase copy number (with one copy of β-tryptase).

### Association analysis

The family-based association test (FBAT, version v2.0.2c, distributed by Harvard University School of Public Health, available at http://www.biostat.harvard.edu/˜fbat/fbat.htm) was used to test association with the series of phenotypic scores described 34,35 under the additive model. This software package was designed for implementing tests of association when the study design is based on families rather than population data. FBAT considers the transmission of alleles from parents to affected offspring and tests for a significant association between the allele and the phenotype of interest; with a dichotomous phenotype, FBAT reduces to the transmission disequilibrium test 36. The FBAT has been applied previously in the same population and has allowed identification of novel asthma genes and genetic associations with patient phenotypes 37,38. The default in FBAT was to use the additive model as several studies have shown that the additive model performs well even when the true genetic model is not additive 34. FBAT output gave for each allele (in this case copy number), the number of informative families for this allele and a test statistic together with the corresponding *Z* score and *P*-value for the test statistic. The association between the copy number and phenotype was considered to be significant if the *P*-value for the test statistic was < 0.05. As several variables were related, tests for multiple variables such as that of the Bonferroni–Holm procedure were not applied to the data presented.

## Results

### Genotyping

Initially, the PCR-RFLP assay was used to genotype DNA samples from the European Collection of Cell Cultures (ECACC) HRC-1 DNA random controls to establish the population frequency of α-tryptase in the UK Caucasian population. It was found that 29.6% of the samples were α-tryptase deficient. The digest method allowed the presence or absence of the α-tryptase allele to be detected in an individual, but did not permit quantification of copy number. When an optimized capillary electrophoresis technique was applied to samples, no α-tryptase alleles not containing the 10- to 11-bp deletion were detected in 96 samples, suggesting the frequency of any such allele was < 0.5% in our population.

**Figure 1:**
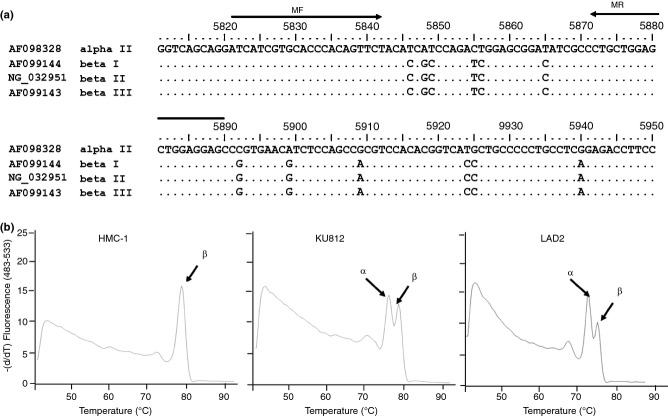
Genotyping assay development. (a) Comparison of α- and β- tryptase nucleotide sequences for the qPCR melting assay. Nucleotide numbers and GenBank accession numbers are indicated. MF, melting forward, MR, melting reverse primers. Six-base pair differences are shown. (b) Melt curve of DNA from the cell lines HMC-1 (β-tryptase alone), KU812 and LAD2 cells (both with α- and β- tryptases).

For the development of a high-throughput system for genotyping, an amplicon was designed to amplify a 73-bp region of the α- and β-tryptase genes. While predicted amplicon length was identical between α- and β-tryptase genes (to achieve equal amplification efficiencies), amplicons differed at six nucleotide positions (including the *Eco*RV restriction site; Fig. 1a). This allowed differentiation on the basis of altered melting profile and restriction, and using the Tm calling module of the LightCycler 480 software, it was possible to define two peaks. With DNA from HMC-1 and KU812 cells as a reference (as these cells express only β-tryptase or a mixture of both α- and β-tryptases, respectively 32), it was possible to distinguish clearly between α- and β-tryptase peaks (Fig. 1b). The LAD2 cell line was found to express both tryptases.

### Cloned artificial controls

**Figure 2:**
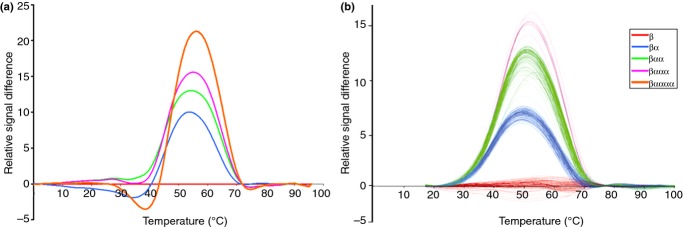
Genotyping of asthma family cohort for α-tryptase copy number. High-resolution melting analysis of possible α-tryptase ratios using (a) cloned PCR DNA fragments, and (b) DNA from the asthmatic family cohort. Difference plots are normalized to the β-tryptase only genotype, and four consistent plots were identified in DNA samples.

To gain information on α-tryptase copy number (as opposed to the simple presence or absence of α-tryptase), positive controls were generated for the melt curve assay that could be mixed in defined ratios to create controls for different numbers of α-tryptase alleles. It was readily apparent that template mixtures containing different ratios for α- and β tryptases gave distinct profiles using the difference plot of the melt curve analysis using a β-tryptase only template as a reference (Fig. 2a). Using the melt curve assay, 1455 individuals in the asthma family cohort were genotyped (Table 1; Fig. 2b). In comparison with the template ratio controls, the majority of cases had either 0, 1 or 2 copies of the α-tryptase gene, suggesting that there is simple copy number variation with 0 or 1 copy of the allele in the population. However, there were a few individuals with different melt profiles raising the possibility that there may be other forms of the tryptase genes. Comparison of these melt profiles to those of the control templates (Fig. 2a) indicated that these individuals carried 3 copies of the α-tryptase gene, suggesting that these individuals carried an allele involving duplication of the α-tryptase gene in addition to a single-copy allele. Genotypes of subjects from the same nuclear families were consistent with this model. The data from the unrelated subjects (parents, *n* = 681) indicated that the frequencies for 0, 1 and 2 copy α-tryptase alleles were 57, 31 and 11%, respectively, with no gender-related differences.

**Table 1:** Clinical characteristics of the asthma family cohort

	Pedigrees (*n* = 1508)[Table-fn tf1-2]	Parents (*n* = 681)	Non-asthmatic parents (*n* = 492)	Asthmatic parents (*n* = 189)	Sibling 1 (*n* = 341)	Sibling 2 (*n* = 328)
Age (year), mean	24.6	40.5	40.7	40.2	13.0	9.9
Gender (% male)	51.8	49.9	51.0	47.1	56.9	53.6
Asthma (% doctor-diagnosed)	60.1	27.8	0.0	100.0	100.0	100
Eczema (% questionnaire)	45.6	32.7	25.8	50.8	57.8	62.4
Hayfever (% questionnaire)	48.9	46.8	38.0	69.8	64.2	47.0
FEV1 (% predicted), mean	98.1	100.8	103.4	94.1	94.7	95.6
BHR (methacholine) (1/L slope + 30) × 1000	19.0	24.3	26.8	17.2	14.6	12.0
Log IgE ([Table-fn tf1-3]Age-corrected)	1.3	0.64	0.49	1.0	1.8	1.9

BHR, bronchial hyperresponsiveness and FEV1, forced expiratory volume in 1s.

Where data were missing for certain individual subjects, they were excluded from subsequent analysis.

Total IgE was measured (kilo units (kU)/L). Log total IgE levels represent the mean log of the standard deviation from the median for each of the following age groups (≥ 5 and ≤ 10, ≥ 10 and ≤ 15, ≥ 15 and ≤ 18, ≥ 18).

### FBAT analysis for α-tryptase copy number variation and asthma and asthma-related phenotypes

Family-based association tests analysis was performed for α-tryptase copy number variation and asthma and asthma-related phenotypes.

The one copy allele was found by FBAT analysis to be associated with lower serum total IgE levels (*P *=* *0.02; Table 2). Data for age-adjusted serum total IgE levels are presented (as in a previous study with this cohort 29), although the same relationships were found also when IgE levels were not corrected for age (data not shown).

**Table 2:** Summary of the association analysis for different numbers of α-alleles and the available asthma phenotypes

Phenotype	α-tryptase haplotype	Allele frequency	Number of informative families	*Z*	*P*
Asthma	0	0.57	271	0.92	0.36
	1	0.31	255	−1.81	0.07
	2	0.11	120	1.2	0.23
Total IgE	0	0.57	271	1.03	0.28
	1	0.31	255	−2.39	0.02
	2	0.11	120	1.92	0.05
Specific IgE dust mite	0	0.57	208	0.45	0.65
	1	0.31	197	−1.96	0.04
	2	0.11	98	2.16	0.03
Specific IgE grass	0	0.57	200	0.63	0.53
	1	0.31	194	−1.49	0.14
	2	0.11	88	1.28	0.20
Specific IgE tree	0	0.57	125	1.17	0.24
	1	0.31	120	−1.78	0.07
	2	0.11	56	0.76	0.44
Atopy severity	0	0.57	245	0.47	0.63
	1	0.31	233	−1.86	0.06
	2	0.11	111	1.98	0.04
Asthma severity	0	0.57	270	0.9	0.36
	1	0.31	255	−1.59	0.11
	2	0.11	122	0.84	0.39
FEV_1_	0	0.57	272	1.02	0.30
	1	0.31	257	−1.91	0.05
	2	0.11	123	1.11	0.26
BHR	0	0.57	264	1.19	0.23
	1	0.31	247	−1.72	0.08
	2	0.11	117	0.51	0.60

Where associations occurred, the *Z* score indicates the direction of the association (+ indicates the allele was over-transmitted (risk), and − indicates it was under-transmitted (protection with respect to asthma affection). The *Z* score is a measure of transmission equilibrium under the null hypothesis (no association, no linkage).

BHR, bronchial hyperresponsiveness and FEV_1_, forced expiratory volume in 1s

Serum levels of IgE specific for mite allergen were found to be associated with both one and two copies of α-tryptase, but this was not found with IgE specific for grass or tree allergens (Table2). The proportions of those with raised levels of IgE specific for dust mite were 48%, for grass pollen 54% and for tree pollen 26%. There was no apparent difference in α-tryptase copy number between those cases for which there was monosensitization or multiple allergen sensitivity as reflected in specific IgE levels. Moreover, there was an apparent trend towards association with lower atopy severity scores (*P *=* *0.06) and reduced susceptibility to asthma (*P *=* *0.07). In contrast, the one copy was associated with lower FEV_1_ (*P *=* *0.05) and an apparent trend towards increased BHR (*P *=* *0.08). There was no apparent association with asthma severity. Analysis of the independent (non-related) parental samples indicated that mean serum IgE levels in those carrying a single copy of the α-tryptase allele (i.e. those with a genotype of 1-α or 2-α) compared with those carrying a null (no copy) α-tryptase allele (i.e. genotype of null-α) were not significantly different. There was no difference in α-tryptase copy number between non-allergic parents (no allergic disease recorded) and those with allergic conditions (with a history of asthma, eczema or hayfever) using Pearson chi-square test (two-sided).

## Discussion

The present study provides evidence that α-tryptase copy number variation is more complex than previously thought and for the first time indicates an association with asthma-related phenotypes. Use of a novel semiquantitative melt curve assay for α-tryptase genotypes has provided evidence that expression of α- and β-tryptase at the locus may be different from that postulated previously; and we have identified individuals carrying alleles containing one, two or for the first time three copies of the α-tryptase gene, as well none at all. Carrying a single copy of the α-tryptase allele was significantly associated with lower serum levels of total and dust mite-specific IgE, and worse lung function, yet there was not a simple relationship between copy number and phenotype. Possessing two copies of the allele were associated with higher serum levels of total and dust mite-specific IgE and increased atopy severity.

The present studies challenge previous proposals for the likely architecture of the α/β-tryptase locus. The application of high-resolution melting analysis for copy number variation resulted in a robust, high-throughput assay, with an error rate of < 1% based on repeated genotyping of a proportion of subjects and analysis of inheritance errors in the family cohort. The finding that individuals can carry 0, 1, 2 or even 3 copies of the α-tryptase gene, would argue against there being a single locus exclusively for α-tryptase 21,23, or a single locus at which there may be allelic competition for α- and β-tryptases with another exclusively for β-tryptases 19,22. Although further studies of β-tryptase copy number will be required to provide definitive information on the structure of that region, there seem to be at least two loci at which genes for α- and β-tryptases compete allelically. Potential genotypes could thus be ββββ, αβββ, ααββ, αααβ or αααα, although the failure to detect the latter would suggest that expression of β-tryptase (unlike α-tryptase) is essential for life. The melting point temperature data are consistent with the idea that copy number variation of the α-tryptase locus is present and accounts for most of the variation observed, although with some heterogeneity in responses we cannot exclude the possibility that multiple α-tryptase loci may exist (as with β-tryptase). It seems likely that copy number variation will contribute substantially to genetic variation in the tryptase gene.

Recognition that large numbers of the general population lack α-tryptase prompted speculation that its presence or absence would affect susceptibility to disease 22, but this is the first investigation of α-tryptase copy number in asthma or allergic conditions. The observed frequency of the α-tryptase-deficient genotype in the population (57%) was higher in the UK population than that observed previously (45%) in a smaller sample of Caucasians in the USA 22. Whether or not this reflects a true difference will require further replication in additional cohorts. Previously in a study of 106 healthy subjects, levels of plasma tryptase were found to be slightly greater in patients expressing α-tryptase than in those without 39, although assays for tryptase available to date fail to distinguish between α and β isoforms 39. Subsequently, in 31 patients with mastocytosis, it has been reported that α-tryptase deficiency was without effect on circulating tryptase levels or clinical severity in mastocytosis 40. Serum was not available for measurement of tryptase levels in the present study, but in this well-characterized cohort of 1350 subjects from 341 asthmatic families that we have examined, α-tryptase copy number was found to be associated with several characteristics of relevance to asthma and allergic disease.

The strongest and perhaps most surprising association was that subjects with a single copy of α-tryptase had significantly lower levels of total serum IgE as well as of IgE specific to dust mite. This was not reflected in lower levels of IgE specific for grass or tree pollen allergens (and at least in the case of the former, this difference cannot be attributed to a lower proportion of those affected with these specific allergies). The precise mechanism whereby copy number is associated with IgE levels is unclear. While α-tryptase lacks enzymatic activity 20,41, one cannot rule out the possibility that tryptases may, like other proteases, have biological actions distinct from catalysis. Another possibility is that the effect is a consequence of altered expression of β-tryptase, or of linked genes nearby rather than the direct inhibitory actions of α-tryptase. β-Tryptase has been implicated in the degradation of IgE 42 rather than its generation, although a feedback process could possibly be involved *in vivo*. The underlying cellular processes require investigation, and of potential relevance is the finding that deficiency in protease-activated receptor 2, a putative target for tryptase 43, is associated with reduced serum levels of allergen-specific IgE in a mouse model 44. Consideration should be given also to the potential for tryptase to stimulate increased IgE levels through impairment of epithelial permeability barrier function (as proposed with filaggrin gene defects 45). It is of interest also that a polymorphism in the promoter region of the gene for chymase, a protease costored with tryptase, has been found to be associated with serum total IgE levels in adult atopic dermatitis in the same large UK Caucasian family cohort 46. Moreover, IgE levels in a mouse model of atopic dermatitis can be suppressed by administration of an inhibitor of chymase, and chymase itself has been reported to increase IgE production from cultured mouse B lymphocytes 47.

The association of α-tryptase genotypes with serum IgE levels in this study did not show a direct gene dosage effect, although for high copy number for α-tryptase, there were many fewer cases. There was the paradoxical finding that possessing one copy of the α-tryptase allele was associated with lower IgE levels, but two copies were associated with higher levels. This was reflected also in atopy severity scores, which were derived in part from IgE measurements in which there was a trend for lower values with one α-tryptase copy, but significantly higher scores with two copies. This would suggest the possibility that α-tryptase copy number variation alleles are acting as proxy markers for variation at or near the β-tryptase locus, through linkage disequilibrium. The adjacent β-tryptase locus appears to have an equally complex structure with significant variation 48. A further layer of complexity is provided by the report of tryptase splice variants for tryptase genes 49 and for a form of β3-tryptase, which like α-tryptase is postulated to be enzymatically inactive 48. The presence of these inactive variants might be related to the copy number variation but this requires further study.

While there was a trend for carrying a single copy of α-tryptase to be protective of asthma susceptibility, this did not reach significance. There was no association with asthma severity in this population which has been used previously to identify new asthmatic genes 31,37,38. It was comprised mostly of those with mild symptoms (as reflected by the mean values of FEV_1_ derived for the population), although there was considerable variation with FEV_1_ values as low as 29 recorded. However, there was a significant inverse association with FEV_1_ measurements and a trend for higher bronchial hyperresponsiveness. As was the case with serum total IgE levels, in all of these analyses, no additive effects were seen with increased copy number.

Mouse tryptases differ substantially in sequence, structure and distribution from those in humans 1 and mice lack α-tryptase 23, but it is of interest that the tryptases reside in a region that was one of the three that was identified in a genome-wide study of loci in this species linked to hyperresponsiveness to methacholine 50. A role for tryptases in bronchial constriction and hyperresponsiveness has been suggested by studies involving the application of tryptase to bronchial explants *in vitro*, or to animal models *in vivo*, and would be in keeping with the potential of tryptase to act directly on airway smooth muscle cells and other structural cells of the airways in ways that would implicate this protease in remodelling processes 1. Moreover, inhibitors of tryptase have been effective in reducing or abolishing early- and late-phase allergen-induced bronchoconstriction in animal models 11–13 and asthmatic subjects 14 and at least in the animal models to decrease airway hyperresponsiveness.

There does not seem to be a straightforward relationship between expression of α-tryptase and phenotypic changes in bronchial asthma, and there is a need for further study and for the present findings to be replicated in independent populations. There are multiple genes involved and it would be premature to propose genetic profiling of individual asthmatic or allergic subjects on the basis of such association studies. However, the significant relationships found between α-tryptase copy number and serum levels of total IgE, atopy severity and lung function measurements in this large UK asthma cohort underscore the potential for tryptases to be important mediators of the allergic tissue response associated with asthma.
